# Determination of Age- and Sex-Specific 99th Percentile Upper Reference Limits for High-Sensitivity Cardiac Troponin I in Healthy Chinese Adults

**DOI:** 10.1159/000523721

**Published:** 2022-02-25

**Authors:** Bing He, Kaijin Wang, Panyang Xu, Qi Zhou, Jiancheng Xu

**Affiliations:** ^a^Department of Laboratory Medicine, First Hospital of Jilin University, Changchun, China; ^b^Department of Clinical Laboratory, Guangzhou Women and Children Medical Center, Guangzhou Medical University, Guangzhou, China; ^c^Department of Pediatrics, First Hospital of Jilin University, Changchun, China

**Keywords:** High-sensitivity, Cardiac troponin I, 99th percentile, Upper reference limits, Reference population

## Abstract

**Introduction:**

Age- and sex-specific reference intervals (RIs) for high-sensitivity cardiac troponin I (hs-cTnI) are not available in China. The objective of the present research was to determine the assay-specific 99th percentile upper reference limits (URLs) for hs-cTnI in healthy Chinese adults.

**Methods:**

Apparently healthy individuals were first screened with a questionnaire. The reference population was selected according to the International Federation of Clinical Chemistry (IFCC) criteria using surrogate biomarker for diabetes, myocardial dysfunction, and renal dysfunction. The serum concentration of hs-cTnI was measured using the automatized VITROS 5600 Immunodiagnostic system (Ortho Clinical Diagnostics, Raritan, NJ, USA).

**Results:**

A total of 2,183 healthy individuals (1,051 men and 1,132 women aged from 20 to 95 years) were enrolled in the study. The serum overall 99th percentile URLs of hs-cTnI were 11.1 ng/L (12.5 ng/L in men, 9.6 ng/L in women). In both men and women, the 99th percentile URLs were significantly higher in individuals ≥55 years old than those in the <55 years old, especially in women. Moreover, 78.8% of males and 70.1% of females presented with hs-cTnI concentrations above the limit of detection of 0.43 ng/L.

**Conclusion:**

The hs-cTnI-VITROS assay coincided with the performance standard of the IFCC for high-sensitivity cTnI assays. The 99th percentile URLs for hs-cTnI in healthy Chinese adults were different from the manufacturer declared and appeared heterogeneous, potentially susceptible to factors such as age and sex.

## Introduction

Myocardial infarction (MI) is delimited as the appearance of acute myocardial injury determined by abnormal cardiac biomarkers in the context of acute myocardial ischemia from the fourth universal definition of MI [1]. Cardiac troponins are sensitive markers of myocardial necrosis and biomarkers of choice for the diagnosis of MI [2]. The high-sensitivity cardiac troponin I (hs-cTnI) test is the latest generation of the cardiac enzyme testing that allows for detection of very low levels of troponins. The International Federation of Clinical Chemistry (IFCC) considers cardiac troponin I “high-sensitive” if: (1) the percentage coefficient of variation at the 99th percentile upper reference limit (URL) does not exceed 10%; (2) measurable cTn is beyond the limit of detection (LoD) in at least 50% of healthy subjects in females and males [3].

The troponin concentration above the 99th percentile indicates myocardial injury; therefore, it is essential to be established on a population selected appropriately. Currently, there is no formal consensus of what defines the reference population [4, 5], and there is an ongoing debate regarding updated American Association for Clinical Chemistry/IFCC criteria [3, 6]. For establishing reference intervals, a questionnaire-based method is recommended from EP28-A3c issued by the Clinical and Laboratory Standards Institute [7], however, that may not exclude all subjects with medical conditions affecting troponin levels. The International Federation of Clinical Chemistry Task Force on Clinical Applications of Cardiac Biomarkers (IFCC TF-CB) recommends additional criteria for defining the 99th percentile URL of cTnI, including the use of surrogate biomarkers for diabetes (glycated hemoglobin [HbA1c]), myocardial dysfunction (natriuretic peptides) and renal dysfunction (estimated glomerular filtration rate [eGFR]); a diverse population, representative of the patient population served, with at least 300 males and 300 females sample size [4, 8]. Consequently, URLs of the same assay can be different, depending on how to select the reference population; the selection method of standardization will be advantageous [4, 9]. The 99th percentile URL may thus depend on demographic characteristics (especially age and sex), physiological variables of individual enrolled and the analytical performances of the implemented assay [4, 5]. Therefore, it is essential to calculate the 99th percentile URLs of every assay in a reference cohort representing the intended use population [10]. There are no studies establishing the 99th percentile URLs in Chinese adults.

This research aimed to assess the sex- and age-specific 99th percentile URLs of hs-cTnI through the VITROS® Immunodiagnostic Products high-sensitive troponin I assay (hs-cTnI-VITROS). Moreover, we analyzed the ability of the assay to detect cTnI between the LoD and the 99th percentile in healthy Chinese adults.

## Materials and Methods

### Study Population and Inclusion/Exclusion Criteria

Healthy subjects aged between 20 and 95 years old were screened through a health questionnaire at the First Hospital of Jilin University between March and July 2020. Subjects who reported no cardiovascular disease or medication use performed an electrocardiogram (ECG), a physical examination, and laboratory testing. Exclusion criteria included pregnancy, a recent blood transfusion or blood donation, hypertension, individuals under 20 years old, HbA1c >6.5% (alternative biomarker of diabetes), eGFR ≤60 mL/min/1.73 m^2^ (alternative biomarker of renal dysfunction), N-terminal-pro-B-type natriuretic peptide (NT-proBNP) >125 pg/mL (<75 years) or 450 pg/mL (≥75 years) (alternative biomarker of myocardial dysfunction), overweight or obesity (body mass index ≥25.0 kg/m^2^), and the history of dyslipidemia or TG >2.3 mmol/L, total cholesterol >6.2 mmol/L, low-density lipoprotein >4.1 mmol/L, and high-density lipoprotein ≤1.0 mmol/L [11]. According to the principles of Helsinki declaration, this study was approved by the Ethics Committee of the First Hospital of Jilin University. All participants signed an informed consent.

### Specimens

At the moment of blood sampling, healthy participants had been maintaining regular diet and physical exercise for the previous 3 days and had been fasting for at least 8 h. Venous blood was collected in vacuum tubes (Vacutainer® SST; Becton Dickinson, Meylan, France) and stored at room temperature for 30 min. All blood specimens were centrifuged at 3,000 rpm for 10 min, and assays were performed within 2 h.

### Analytical Evaluation

Serum hs-cTnI levels were measured using the hs-cTnI-VITROS assay (Ortho Clinical Diagnostics, Pencoed, UK). According to the manufacturer, the overall serum 99th percentile URL of hs-cTnI corresponded to 11 ng/L (9 ng/L in females and 12 ng/L in males) with a corresponding coefficient of variation <7% at the 99th percentile. The LoD, limit of blank, and limit of quantification were 0.43 ng/L, 0.23 ng/L, and 1.23 ng/L, respectively. The measurement precision was assessed using the four concentration samples by multiple dilutions, in 3 replicates each day for a total of 5 days, according to EP5-A2 guideline by Clinical and Laboratory Standards Institute [12].

### Other Laboratory Tests

NT-proBNP was measured using the VITROS Immunodiagnostic Products NT-proBNP assay on the VITROS 5600 Immunodiagnostic System (Ortho Clinical Diagnostics, Pencoed, UK). HbA1c was determined by ion-exchange High Performance Liquid Chromatography using the Tosoh HLC-723G8 analyzer (Tosoh Corporation, Tokyo, Japan). The eGFR was calculated by the Chronic Kidney Disease-Epidemiology Collaboration equation. Other biochemical parameters were analyzed through the Hitachi 7600-210 automatic analyzer (Hitachi High-Technologies, Tokyo, Japan). All laboratory procedures were performed by trained laboratory staff. The laboratory of the First Hospital of Jilin University was accredited the ISO 15189 Medical Laboratories certification-special requirements for quality and ability of China National Accreditation Service for Conformity Assessment.

### Statistical Analysis

SPSS Software version 19.0 (SPSS Inc., Chicago, IL, USA), MedCalc Software version 15.6 (MedCalc Software, Ostend, Belgium), and R Software version 3.2 were used for statistical analysis. The Kolmogorov-Smirnov test was used to investigate the normal distribution of data. Dixon method was utilized to distinguish outliers from the normal subjects [7]. After excluding the outliers, nonparametric methods were used to calculate the 99th percentile URLs according to the EP28-A3c [7]. The levels of hs-cTnI between two subgroups were compared by the Mann-Whitney U test. Association between variables was investigated by the Spearman correlation test. A *p* value <0.05 was considered statistically significant.

## Results

### Characteristics of the Study Population

**Fig. 1:**
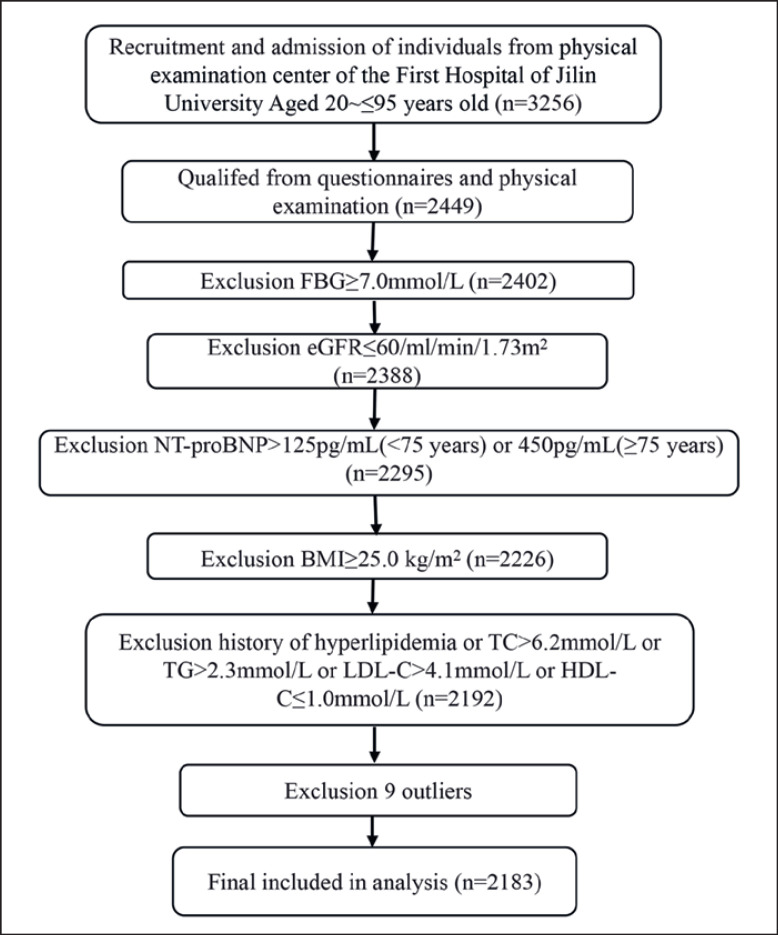
Flow diagram for selection of the individuals on establishing 99th percentile URL of hs-cTnI.

The flow diagram of participants' enrollment is shown in Figure 1. Of the 3,256 screened adults, 807 were excluded due to the presence of medical conditions detected by the questionnaires or physical examination. Of the remaining 2,449 subjects, 61 were removed due to abnormal HbA1c or eGFR values. Additional 93 subjects were removed for abnormal NT-proBNP. Subjects with body mass index ≥25.0 kg/m^2^ (*n* = 69) and hyperlipidemia (*n* = 34) were also excluded. On the basis of Dixon method, 9 hs-cTnI values were removed as outliers. A total of 2,183 healthy adults (mean age 54.2 ± 19.5 years, range 20–95 years) were enrolled in the study. They were 1,051 males (mean age 54.2 ± 19.8 years) and 1,132 females (mean age 54.2 ± 19.2 years). Of the 2,183 participants, 1,106 (51%) were less than 55 years old (539 males and 567 females). Detailed demographic characteristics and laboratory results are revealed in Table 1.

### Distribution and 99th Percentile URL for Hs-cTnI

**Fig. 2:**
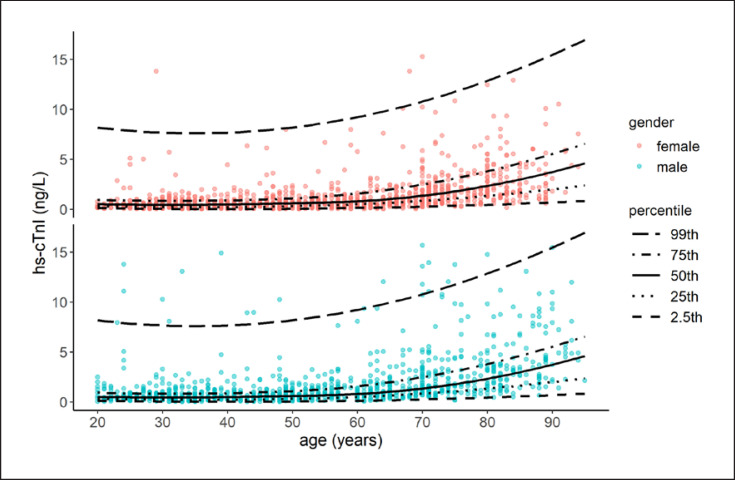
Age- and sex-specific scatter plots of hs-cTnI (20∼≤95 years).

**Fig. 3:**
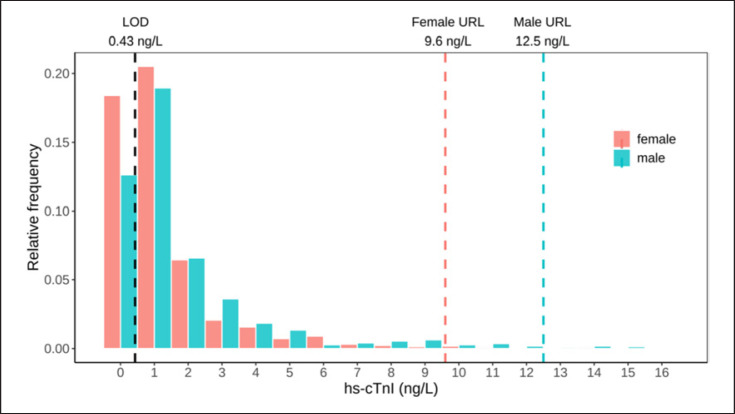
Relative frequencies according to sex for hs-cTnI.

Figure 2 shows the scatter plots of hs-cTnI by age and sex distribution. The median concentration of hs-cTnI in all subjects was 0.8 ng/L (0.9 ng/L in men and 0.7 ng/L in women). The median level of hs-cTnI in ≥55 years old (1.5 ng/L) was higher than in <55 years old (0.5 ng/L) (*p* < 0.001). In both age groups, the median concentration of hs-cTnI was higher in males than in females. The median and 99th percentiles of hs-cTnI concentrations for all participants, as well as with the age and sex difference, are shown in Table 2. Relative frequencies of hs-cTnI according to sex are presented in Figure 3. As shown in Figure 3, the distribution of hs-cTnI levels in the study population was severely skewed. The 99th percentile URL of hs-cTnI for all participants was 11.1 ng/L (90% CI: 10.3–12.9 ng/L). The 99th percentile URL was distinctly higher in males (12.5 ng/L, 90% CI: 11.1–13.8 ng/L) than in females (9.6 ng/L, 90% CI: 7.6–10.9 ng/L) (Fig. 3). The overall 99th percentile URL in ≥55 years old (12.6 ng/L, 90% CI: 11.1–13.8 ng/L) was higher than in <55 years old (8.0 ng/L, 90% CI: 5.0–10.3 ng/L). The 99th percentile URL was 10.3 ng/L in males <55 years old, while it was 13.7 ng/L in males ≥55 years old. In females, the 99th percentile URL was 5.3 ng/L in <55-year-old group and 10.6 ng/L in ≥55-year-old group. The sex difference of hs-cTnI level was most obvious in the <55-year-old group.

### Correlation Analysis

**Fig. 4:**
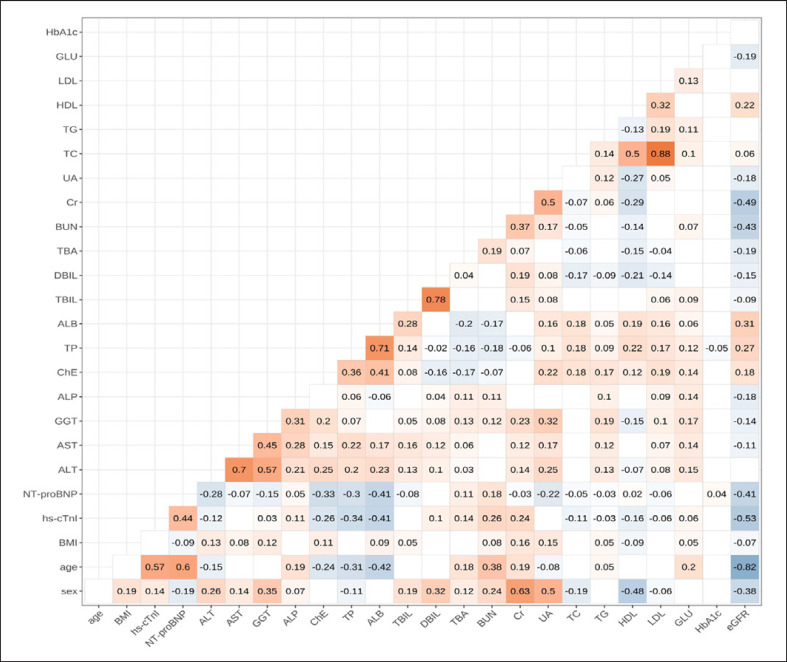
Spearman correlation heat map among 25 variables in the healthy population aged 20∼≤95 years.

The hs-cTnI values were significantly correlated with age (*r* = 0.57, *p* < 0.001) and sex (*r* = 0.14, *p* < 0.001). Our study also analyzed correlations among different biomarkers and demonstrated a strong correlation between hs-cTnI and NT-proBNP (*r* = 0.44, *p* < 0.001). Significant negative correlations (*p* < 0.001) were presented between hs-cTnI and eGFR, albumin and total protein. The correlation analysis is displayed in Figure 4.

### High-Sensitivity Designation of the Hs-cTnI Assay

The percentage of hs-cTnI measurable values ≥ LoD (0.43 ng/L) are provided in Table 3. Overall, 74.3% of healthy participants (78.8% of males and 70.1% of females) showed higher hs-cTnI concentrations than LoD. Rates of detectable values were gradually increased with age. Within imprecision and total imprecision were 3.6% and 4.2%, respectively, at the 99th percentile URL.

### Comparison with Previous Evaluations

The detection system, mean ages, and sex-specific URLs reported by the manufacturer and previous studies are presented in Table 4. The 99th percentile URLs of hs-cTnI in males were higher than in females in all previous studies. The 99th percentile URLs of hs-cTnI of this study were different from the manufacturer's recommendation and lower than the URLs from previous studies.

## Discussion

As we know, the present study reported the 99th percentile URLs of hs-cTnI-VITROS assay in a large sample of healthy Chinese adults for the first time. Both age and sex influenced the 99th percentile URLs. Although the sex-specific 99th percentiles in clinical utility were controversially discussed [13, 14, 15], the IFCC TF-CB [16] acknowledged sex-specific 99th percentiles to avoid underestimation of MI by the general 99th percentiles [1, 3]. From a clinical point of view, compared with the overall 99th percentile of the mixed population of both sexes, a lower URL for females increased clinical sensitivity at the expense of clinical specificity, while the opposite was true for males, and the false-positive rate of MI diagnoses in females increased significantly [17]. For risk stratification, researches had proved that using threshold values below either the women or overall 99th percentile URLs enhanced prediction [18].

Our results indicated that the levels of cTnI were increased in both sexes after 55 years old. Previous population-based studies indicated that hs-cTnI levels increased with age [19]. This could be due to a number of factors, including the presence of comorbidities [16]. There is currently no consensus on the clinical utility of age- or ethnic-specific 99th percentile URLs. A single study showed that African-Americans had higher 99th percentiles than Caucasians [20]. Of interest, the presence of multiple screening criteria seems to be associated with a lower 99th percentile URL of hs-cTnI [4, 21]. In our study, we used more screening criteria, showing a lower 99th percentile URL of hs-cTnI than Koreans [22], Pakistanis [23], and Germans [24]. Other studies indicated that cTnI levels may vary with age, ethnicity, population size, inclusion/exclusion criteria, and the statistical analysis approach utilized for calculating the 99th percentile [5, 25]. As we only focused on the Chinese population in our study, we could not observe ethnic discrepancy in the 99th percentile URLs of cTnI assays in the present study.

Previous to this study there were no 99th percentile URLs established in an Asian population using the hs-TnI-VITROS assay. We showed lower cTnI values in males than those reported in a US study [26]; on the contrary, the cTnI values in females were higher (9.6 vs. 5.0 ng/L). The difference might be related to age, ethnicity, or population size (2,183 vs. 694). In another Chinese study using the Abbott hs-cTnI assay [27], the 99th percentile URLs were higher than our value. The distinct study populations examined with different assays might explain the inconsistent results.

It was extremely essential for clinicians to know the characteristics of the various cTn assays as patients could be transferred from one hospital to another and the cTn methods might differ between sites. One method to assess sensitivity of the hs-cTn assays was by measuring the percentage of healthy individuals with a cTnI value above the LoD of the assay. The IFCC TF-CB had recommended the LoD as the minimum reportable limit for detecting a hs-assay definition [8]. Most, though not all, high-sensitivity assays meet the joint Academy/Clinical Application of Cardiac Bio-Markers guidelines [3], which included measurable concentrations ≥ LoD in >50% of healthy subjects, particularly for females. Giannitsis et al. [24] found that the Abbott assay detecting cTnI did not satisfy the requirements of a high-sensitivity assay. A more recent Opinion Paper [2] stated that high-sensitivity assays evaluating cTnI should meet at least two criteria that were accomplished by our study. In addition, we met the criteria endorsed by the International Federation of Clinical Chemistry Task Force on Clinical Applications of Cardiac Biomarkers [3].

### Strengths and Limitations

Our study enrolled a large sample of healthy Chinese individuals with a wide age range (20–95 years), following the latest guidelines for defining the 99th percentile URL in males and females. Compared with previous criteria, we strengthened the selection criteria by specifically excluding people who were overweight or obesity, or with a dyslipidemia.

This study has also several limitations. First, we did not perform an echocardiogram or magnetic resonance imaging. Although ECG cannot replace echocardiogram or magnetic resonance imaging in the diagnosis of cardiovascular diseases, we thought that ECG combined with NT-proBNP could be sufficient to identify healthy individuals. Second, the implemented LoD originated from the manufacturers' instructions, it was not independently measured. Third, we did not calculate the 99th percentile URL values with different statistical approaches.

## Conclusion

This was the first evaluation of VITROS Immunodiagnostic Products hs Troponin I Reagent in an age- and sex-matched Asian population. Our findings confirmed that the 99th percentile URLs could be influenced by age, sex, and the screening criteria to define the healthy population. In the current study, the 99th percentile URLs were significantly higher in ≥55 years old than in <55 years old, especially in females. Establishing the 99th percentile URL for the hs-cTnI-VITROS assay specific might improve clinical decision-making and health outcomes in the Chinese population.

## Statement of Ethics

This study complied with the Declaration of Helsinki. This study protocol was reviewed and approved by the Ethics Committee of the First Hospital of Jilin University, approval number (20K021-001). All subjects gave their written informed consent for participation.

## Conflict of Interest Statement

The authors have no conflicts of interest to declare.

## Funding Sources

This work was supported by grants from Jilin Science and Technology Development Program (Grant No. 20190304110YY, 20200404171YY), Scientific and Technological Project of Jilin Provincial Department of Education (Grant No. JJKH20211177KJ), and the First Hospital Translational Funding for Scientific & Technological Achievements (Grant No. CGZHYD202012-005).

## Author Contributions

Bing He: formal analysis, investigation, and writing − original draft. Kaijin Wang: formal analysis, review, and editing. Panyang Xu: review and editing. Qi Zhou: project administration and resources. Jiancheng Xu: project administration, resources, and supervision.

## Data Availability Statement

All data generated or analyzed during this study are included in this article. Further enquiries can be directed to the corresponding author.

## Figures and Tables

**Table 1:** Baseline characteristics of subjects aged 20~≤95 years (*n* = 2,183)

Analytes	20~<30 yr		30~<40 yr		40~<50 yr		50~<60 yr		60~<70 yr		70~<80 yr		>80 yr		Total	
	M	F	M	F	M	F	M	F	M	F	M	F	M	F	M	F
*N*	132	147	183	173	150	167	134	169	150	160	159	178	143	138	1,051	1,132
BMI, kg/m^2^	23.1	20.9	22.4	21.5	22.5	21.7	22.6	21.6	22.3	22.4	22.3	22.2	22.0	21.3	22.5	21.7
hs-cTnl, ng/L	0.52	0.36	0.52	0.60	0.58	0.53	0.81	0.56	1.05	0.75	1.88	1.50	3.42	2.47	0.94	0.74
NT-proBNP, pg/mL	6.6	19.0	8.4	24.3	13.0	26.3	20.5	33.7	31.5	44.5	55.5	55.4	137.3	161.5	23.9	38.8
ALT, U/L	21.5	12.3	26.4	14.2	20.8	15.0	20.5	17.0	18.7	17.3	16.4	13.7	13.5	12.3	19.2	14.3
AST, U/L	18.8	15.4	20.8	16.4	20.3	17.2	21.1	19.5	19.4	20.4	19.4	18.5	18.1	18.0	19.8	17.9
GGT, U/L	22.2	12.2	29.1	12.4	23.9	13.7	26.6	16.5	25.4	16.8	20.9	17.8	18.1	15.4	23.7	15.1
ALP, U/L	62.9	50.5	62.3	49.5	61.4	53.9	69.3	67.7	66.4	69.8	60.4	67.1	60.2	64.4	62.7	59.9
TP, g/L	73.2	72.8	73.1	73.8	71.6	72.9	71.2	72.9	69.8	72.4	67.8	69.9	63.8	66.0	70.8	72.2
ALB, g/L	44.3	42.1	43.4	41.8	43.1	41.7	42.3	42.6	40.3	41.3	38.2	39.1	35.2	35.5	41.5	41.1
TBIL, µmol/L	13.3	12.0	14.9	11.3	15.1	12.7	14.7	13.3	14.5	12.7	14.5	13.4	13.2	11.6	14.5	12.5
DBIL, µmol/L	3.3	2.9	3.4	2.3	3.4	2.4	3.5	2.6	3.3	2.2	3.4	2.7	3.4	2.4	3.4	2.5
TBA, vmol/L	2.7	3.0	3.6	2.5	2.8	2.6	3.1	2.7	3.7	2.8	4.0	3.4	5.1	3.8	3.6	3.0
BUN, mmol/L	4.5	4.0	5.1	4.2	5.3	4.2	5.3	4.7	5.8	5.0	6.2	5.4	6.4	5.6	5.4	4.7
Cr, µmol/L	68.2	50.0	70.5	52.0	72.5	52.2	66.5	54.6	69.2	57.4	75.3	57.2	72.9	61.0	70.6	54.3
UA, µmol/L	376	284	374	254	347	264	340	264	331	273	332	288	325	272	348	270
TC, mmol/L	4.3	4.2	4.5	4.4	4.4	4.8	4.3	4.9	4.4	4.9	4.2	4.7	3.9	4.6	4.3	4.7
TG, mmol/L	1.2	1.0	1.3	1.1	1.3	1.1	1.2	1.3	1.3	1.4	1.3	1.3	1.1	1.2	1.2	1.2
HDL, mmol/L	1.1	1.3	1.2	1.4	1.2	1.4	1.2	1.4	1.2	1.4	1.1	1.3	1.1	1.3	1.1	1.3
LDL, mmol/L	2.7	2.3	2.7	2.4	2.6	2.7	2.6	2.8	2.6	2.7	2.5	2.8	2.3	2.7	2.5	2.7
GLU, mmol/L	5.0	5.0	5.1	5.0	5.3	5.1	5.6	5.4	5.5	5.5	5.4	5.5	5.3	5.3	5.3	5.2
HbA1c,%	5.3	5.2	5.3	5.3	5.4	5.3	5.3	5.4	5.3	5.5	5.4	5.3	5.3	5.5	5.3	5.3
eGFR, mL/min	131.4	145.9	122.2	136.5	113.5	127.1	108.5	120.0	99.7	110.8	91.6	104.6	85.1	96.4	106.9	122.5

Data presented as *n* (%) or median. BMI, body mass index; hs-cTnl, high-sensitivity cardiac troponin I; NT-proBNP, N-terminal-pro-B-type natriuretic peptide; ALT, alanine aminotransferase; AST, aspartate aminotransferase; GGT, γ-glutamyltransferase; ALP, alkaline phosphatase; TP, total protein; ALB, albumin; TBIL, total bilirubin; DBIL, direct bilirubin; TBA, total bile acid; BUN, urea nitrogen; Cr, creatinine; UA, uric acid; TC, total cholesterol; TG, triglyceride; HDL, high-density lipoprotein; LDL, low-density lipoprotein; GLU, glucose; HbA1c, glycated hemoglobin; eGFR, estimated glomerular filtration rate; M, male; F, female.

**Table 2:** Median hs-cTnl concentrations and 99th percentiles (*n*g/L) by age group and sex

Age group	Total				Males				Females			
	*n*	median	99th percentile (90% CI)	*p* value	*n*	median	99th percentile (90% CI)	*p* value	*n*	median	99th percentile (90% CI)	*p* value
<55 years	1,106	0.5	8.0 (5.0–10.3)		539	0.6	10.3 (8.0–13.8)		567	0.5	5.3 (3.6–8.0)	
≥55 years	1,077	1.5	12.6 (11.1–13.8)	<0.001	512	1.7	13.7 (12.0–15.5)	<0.001	565	1.3	10.6 (9.3–13.8)	<0.001
Overall	2,183	0.8	11.1 (10.3–12.9)		1,051	0.9	12.5 (11.1–13.8)		1,132	0.7	9.6 (7.6–10.9)	

CI, confidence interval; hs, high-sensitivity; cTn, cardiac troponin.

**Table 3:** Percentage of measurable values (≥LoD) of hs-cTnl according to sex and age

Age	Male		Female		All	
	*n*	values ≥LoD,^a^ %	*n*	values ≥LoD,^a^ %	*n*	values ≥LoD,^a^ %
20–29 years	132	60.6	147	45.6	279	52.7
30–39 years	183	59.6	173	56.1	356	57.9
40–49 years	150	64.0	167	59.3	317	61.5
50–59 years	134	82.8	169	60.9	303	70.6
60–69 years	150	86.7	160	75.6	310	81.0
70–79 years	159	98.1	178	93.3	337	95.5
80–95 years	143	100.0	138	99.3	281	99.6
Overall	1,051	78.8	1,132	70.1	2,183	74.3

LoD, limit of detection; hs, high-sensitivity; cTn, cardiac troponin.

a LoD 0.43 ng/L for hs-cTnl.

**Table 4:** Comparison of 99th percentile URL for hs-cTnl with other studies (*n*g/L)

	System	*N*	Mean age ± SD, years	Overall 99th percentile (90% CI)	Male 99th percentile	Female 99th percentile
This study	VITROS 5600	2,183	54.2±19.5	8.0 (5.0–10.3) (<55 years)	10.3 (<55 years)	5.3 (<55 years)
			22–91^b^	12.6 (11.1–13.8) (≥55 years)	13.7 (≥55 years)	10.6 (≥55 years)
Manufacturer^a^	VITROS 5600	952		11.0 (7.8–16.3)	12.0	9.0
Korea [22]	ARCHITECT i2000SR	854	49.8±10.2	18.0 (14.0–35.0)	20.0	19.0
Sichuan, China [27]	ARCHITECT i2000	1,485	36.0±13.0	28.0 (*N*R)	31.1	22.7
US [26]	VITROS 3600	694	40.0±12.0	15.0 (9.0–26.0)^c^	16.0	5.0

SD, standard deviation; CI, confidence interval; NR, not reported; hs, high-sensitivity; cTn, cardiac troponin.

a Manufacturer's data came from VITROS Immunodiagnostic Products hs troponin I Reagent Pack instructions.

b Age range was reported.

c 95% CI was reported.
